# Bradykinin Preconditioning Improves Therapeutic Potential of Human Endothelial Progenitor Cells in Infarcted Myocardium

**DOI:** 10.1371/journal.pone.0081505

**Published:** 2013-12-02

**Authors:** Zulong Sheng, Yuyu Yao, Yefei Li, Fengdi Yan, Jie Huang, Genshan Ma

**Affiliations:** Department of Cardiology, Zhongda Hospital, Medical School of Southeast University, Nanjing, Jiangsu, China; Tokai University, Japan

## Abstract

**Objectives:**

Stem cell preconditioning (PC) is a powerful approach in reducing cell death after transplantation. We hypothesized that PC human endothelial progenitor cells (hEPCs) with bradykinin (BK) enhance cell survival, inhibit apoptosis and repair the infarcted myocardium.

**Methods:**

The hEPCs were preconditioned with or without BK. The hEPCs apoptosis induced by hypoxia along with serum deprivation was determined by annexin V-fluorescein isothiocyanate/ propidium iodide staining. Cleaved caspase-3, Akt and eNOS expressions were determined by Western blots. Caspase-3 activity and vascular endothelial growth factor (VEGF) levels were assessed in hEPCs. For *in* *vivo* studies, the survival and cardiomyocytes apoptosis of transplanted hEPCs were assessed using 1,1′-dioctadecyl-3,3,3′,3′-tetramethylindodi- carbocyanine,4-chlorobenzenesul-fonate salt labeled hEPCs and TUNEL staining. Infarct size and cardiac function were measured at 10 days after transplantation, and the survival of transplanted hEPCs were visualized using near-infrared optical imaging.

**Results:**

*In* *vitro* data showed a marked suppression in cell apoptosis following BK PC. The PC reduced caspase-3 activation, increased the Akt, eNOS phosphorylation and VEGF levels. *In* *vivo* data in preconditioned group showed a robust cell anti-apoptosis, reduction in infarct size, and significant improvement in cardiac function. The effects of BK PC were abrogated by the B2 receptor antagonist HOE140, the Akt and eNOS antagonists LY294002 and L-NAME, respectively.

**Conclusions:**

The activation of B2 receptor-dependent PI3K/Akt/eNOS pathway by BK PC promotes VEGF secretion, hEPC survival and inhibits apoptosis, thereby improving cardiac function *in* *vivo*. The BK PC hEPC transplantation for stem cell-based therapies is a novel approach that has potential for clinical used.

## Introduction

Stem cell-based therapies offer promising approaches to improve cardiac function after myocardial injury. Several studies have suggested that endothelial progenitor cells (EPCs) participate in the process of neovascularization and tissue repair to enhance the recovery of the ischemic myocardium [1,2]. Although stem cell transplantation shows great promise for cardiac reparative and regenerative therapy, the effectiveness of the engraftment and survival of the transplanted stem cells within the ischemic myocardium limits its use. A study reported only a few mesenchymal stem cell (MSC) survivors after 1-week injection of stem cells [3]. Our previous study also showed a limited number of EPC survivor at day 10 post myocardial infarction (MI) [4]. Apoptosis is considered as one of the mechanisms of cell death in the ischemic myocardium [5]. Therefore, strategies to decrease apoptosis are important for cell survival to regenerate cardiac function in the infarcted myocardium.

Preconditioning (PC) of stem cells is a new strategy used to minimize the massive death of cells after transplantation [6]. Ischemic preconditioning (IPC) reportedly enhances the survival of stem cells during transplantation in the infarcted myocardium [7]. The PC of MSCs with diazoxide reportedly promotes the survival and angiomyogenic potential of MSCs in the infarcted myocardium [8]. 

Bradykinin (BK), the main metabolite in the tissue kallikrein-kinin system (KKS), reportedly has an important function in regulating the tolerance of an ischemic heart [9]. BK has been widely accepted as one of the endogenous protective factors for improving cardiac function, and attenuate cardiomyocyte apoptosis after myocardial injury [10]. The effect of BK is mediated by the B1 receptor (B1R) and B2 receptor (B2R) according to the relative potencies and affinities to their agonists [11]. B2R is constitutively expressed in human EPCs (hEPCs), whereas B1R is weakly expressed [12]. BK limits the infarct size in the ischemic heart during the late phase of PC by activating B2R [13]. Furthermore, BK PC can improve left ventricular performance and limit myocardial apoptosis [14]. 

However, the BK PC of stem cells prior to transplantation has not been reported thus far. In this study, we hypothesized that the PC hEPCs with BK will significantly enhance cell survival, inhibit apoptosis, and promote infarcted myocardium repair.

## Materials and Methods

### Ethics Statement

The use of human cord blood in this study was approved by the Medical Ethics Committee of Zhongda Hospital Affiliated to Southeast University (approval ID: 2010 ZDLL05). A written informed consent was obtained from all selected pregnant women involved in the study. All animal studies were performed via a protocol approved by the Institutional Animal Care and Use Committee of Southeast University and complied with the National Research Council’s guidelines (approval ID: SYXK-2010.0510). 

### Isolation, Culture and Characterization of hEPCs

Human umbilical cord blood was obtained from Zhongda Hospital in accordance with the approved institutional review board protocol. The obtained human umbilical cord blood was diluted to a 1:1 ratio in phosphate-buffered saline (PBS). The mononuclear cell (MNC) fraction was obtained from a Lymphoprep density gradient (Sigma) after centrifugation, washed twice in PBS, and centrifuged. The cell pellet was suspended in endothelial basal growth medium (EBM-2) supplemented with EGM-2 MV SingleQuots and 5% heat inactivated fetal bovine serum (FBS) (EGM-2, Lonza, Walkersville, MD, USA). The solution was plated in a T-25 culture ﬂask coated with 10 µg/ml human plasma ﬁbronectin (FN, Millipore, Bedford, MA, USA). After removing unbound cells at 96 hours with the bound cell fraction maintained in culture using EGM-2, spindle-shaped cells were observed after seven days. Colonies of endothelial-like cells grew until confluent, and were trypsinized and plated uniformly in a new T-25 culture ﬂask as the ﬁrst passage. The medium was changed every three days, and at 80% conﬂuence, the cells were passaged at a ratio of 1:2. Subsequent passages were performed similarly, and passages 3 ~ 6 hEPCs were used in the study. 

Cells were primarily characterized by phase contrast microscopy to evaluate the cobblestone morphology. Cells were incubated with 1, 1’-dioctadecyl 3,3,3’,3’-tetra- methylindo-carbocyanine (DiI)-labeled acetylated low-density lipoprotein (LDL) (DiI -acLDL, Invitrogen, Carlsbad, California, USA) for 4 h at 37 °C. Lectin binding was analyzed using fluorescein isothiocyanate (FITC)-conjugated UEA-1-lectin (Sigma), and the cells were examined under a fluorescence microscope (Nikon Corporation). Immunofluorescence was also utilized to determine the expression of the progenitor lineage marker CD34 (BD Biosciences, Bedford, Massachusetts, USA) and the B2R expression (BD Biosciences). Flow cytometry was utilized to analyze the expression of the progenitor lineage marker CD34, the immature hematopoietic cell marker CD133 (Santa Cruz), the endothelial lineage markers VEGFR2 (KDR, BD Biosciences) and CD146 (Santa Cruz), the leukocyte marker CD45 (BD Biosciences), the B1R (BD Biosciences) and B2R expression. 

### Effect of Different Concentrations and Time Points of BK PC on Cell Apoptosis

To mimic ischemic microenvironment *in vitro*, hypoxia was achieved by placing the cells in a Modular Incubator Chamber containing a mixture of 0.1%O_2_, 5%CO_2_ and 94.9%N_2_ (Billups-Rothenberg; Del Mar, CA, USA) according to the manufacturer’s instructions as our previously research described [15]. The hEPCs were cultured with various concentrations of BK PC (1 and 10 nM) for 10 min with RPMI-1640 medium, and then apoptosis was induced by hypoxia along with serum deprivation for 12 h to assess the effect of different concentrations of BK PC on cell apoptosis. The percentage of apoptotic hEPCs in the various concentrations of the treatment groups was determined with the annexin V-FITC/ propidium iodide (PI) apoptosis detection kit (Biouniquer Technology CO., Hangzhou, China) according to the instructions of manufacturer. Fluorescence was detected by flow cytometry (FACSCalibur, BD Biosciences). The apoptotic cells were stained with annexin V, necrotic cells displayed PI staining positive and annexin V negative, whereas live cells remained unstained.

The various time points of BK PC (10 min, 1 h, 4 h and 12 h) were detected by Western blots to analyze the expression of Akt and Phospho-Akt [Ser473] (1:1000, Cell Signaling). The procedures follow the fashion which was similar to the method described previously [8]. 

### Assessment of hEPCs Apoptosis

The hEPCs were grown on six-well plates incubated in EBM-2 supplemented with 1% FBS for 16 h. The hEPCs were then randomly assigned to the following nine experimental groups: the Neg Con (negtive control) group composed of non-PC hEPCs incubated with EGM-2 under normoxia for 12 h; the Pos Con (positive control) group composed of non-PC hEPCs exposed to hypoxia along with serum deprivation for 12 h; the HOE140, LY294002 and L-NAME groups composed of hEPCs incubated with either 150 nM of HOE140 (Sigma) or 10 µM of LY294002 (LY, Sigma) or 100 µM of L-NAME (LN, Sigma) for 30 min independent of BK PC and then treated with hypoxia and serum deprivation for 12 h ; the BK PC group composed of PC hEPCs incubated with 10 nM BK (Sigma, St. Louis, MO, USA) for 10 min and exposed to hypoxia along with serum deprivation for 12 h; and the BK PC/HOE, BK PC/LY and BK PC/LN groups composed of PC hEPCs incubated with either 150 nM of HOE140 or 10 µM of LY294002 or 100 µM of L-NAME, respectively, for 30 min prior to BK, and then treated with hypoxia and serum deprivation for 12 h. In every single step, three times of washes were applied with pre-warmed PBS, in order to remove the residual serum and drugs. 

The percentage of apoptotic hEPCs in each group was determined using annexin V/PI staining analyzed by flow cytometry. 

### Western Blots Analysis

After treatment, the adherent and non-adherent cells were washed twice with PBS, lysed in lysis buffer [10 mmol/L Tris-HCl, pH 7.4, containing 1% Triton X-100, 100 mmol/L sodium chloride, 20 mmol/L sodium pyrophosphate, 2 mmol/L sodium orthovanadate, 2 mmol/L EDTA, and 1% protease inhibitor cocktail (Sigma)], and centrifuged at 12000 g and 4°C for 30 min. Protein concentration was determined using a protein assay kit (Thermo scientific, Rockford, USA), and samples were mixed with SDS-denaturating sample buffer, and separated through 10% SDS-PAGE gels. The proteins were transferred to a PVDF membrane by electrophoresis. The membrane was blocked and incubated overnight on a rocking platform at 4°C with antibodies against Akt, Phospho-Akt [Ser473], Phospho-eNOS [Ser1177], cleaved caspase-3, pro-caspase-3 (1:1000, Cell Signaling, MA, USA), as well as eNOS (1:1000, BD Biosciences) and GAPDH (1:1000 Kangchen Biology Inc., Shanghai, China). Then HRP-conjugated secondary antibodies were incubated for 1 h and exposure with Molecular Imager ChemiDoc XRS System (Bio RAD). Relative intensities of protein bands were analysed by Image-pro plus 6.0 (Media Cybernetics, Silver Spring, MD, USA).

### Measurement of Cell Caspase-3 Activity and Vascular Endothelial Growth Factor (VEGF) Levels

Cell caspase-3 activity was measured using a caspase-3 colorimetric assay kit (KeyGEN Biology Inc., Nangjing, China), according to the instructions of the manufacturer. Briefly, cells after treatment were washed with cold PBS, re-suspended in lysis buffer containing 20 mM Tris-HCl, pH 7.2, 150 mM NaCl, 1% Triton X-100 and 1 mM dithiothreitol (DTT), and left on ice for 20 min. The lysate was centrifuged at 16,000 × g at 4°C for 15 min. Protein content was determined using the Bradford protein assay (Biorad, Hercules, CA). Each sample was adjusted to 200 μg of protein and then incubated for 1 h at 37°C with 10 μl caspase-3 substrate (Ac- DEVDpNA) (2 mM). The absorbance was determined at 405 nm and caspase-3 activity was assessed by calculating the ratio of the OD 405 nm of the drug-treated cells to the untreated cells.

VEGF levels in the cell supernatant was detected using an enzyme-linked immunosorbent assay (ELISA) kit (R&D Systems Inc., Minneapolis, MN, USA) according to the manufacturer’s instructions.

### In Vitro Experiments with the Conditioned Medium

Conditioned medium was generated as follows: (i) non-PC hEPCs incubated with EGM-2 for 12 h (negative control); (ii) non-PC hEPCs incubated for 12 h under hypoxia along with serum deprivation (positive control); (iii) preconditioned with BK for 10 min and incubated for 12 h under hypoxia along with serum deprivation. (iv) incubated with either HOE140 or LY294002 or L-NAME, for 30 min prior to BK, and exposed to hypoxia along with serum deprivation for 12 h. The medium was then collected and used for in vitro experiments. 

Cardiomyocytes were isolated from 1–2-day old neonatal Sprague-Dawley rats (Animal Center, Yangzhou University, China) as previously described [16]. We used a 60-min pre-plating procedure to reduce the number of non-myocytes in the culture. Then, after 3 days in culture, the isolated rat neonatal cardiomyocytes were cultured in supernatant collected from the cultures treated as stated above, and then exposed to hypoxia for 12 h. However, in the Neg Con group, the cardiomyocytes were cultured in the negative control conditioned medium and then exposed to normoxia for 12 h. Additionally, in the BK PC/VEGF Ab group, the conditioned medium of BK-PC-hEPCs was mixed with neutralizing anti-VEGF antibody (VEGF Ab; 500 ng/mL, R & D System) 30min prior to stimulation of the cardiomyocytes [17]. Following incubation, the cells were collected and the number of apoptotic cells was determined using Hoechst 33342 staining. Cells were then examined and immediately photographed under a fluorescence microscope (Nikon Corporation). The apoptotic nuclei of cells were assessed by counting the number of cells that displayed nuclear morphology changes, such as chromatin condensation and fragmentation.

### Nude Mouse Model of Acute MI and Transplantation of Labeled hEPCs

All animal studies were approved by the Institutional Animal Care and Use Committee of Southeast University. The recipient male BALB/C nude mice (20 g to 22 g, weight) were intraperitoneally anesthetized with 45 mg/kg of pentobarbital, intubated, and then ventilated at 110 breaths per min. In the sham group, mice were subjected to sham surgery followed by medium injection. The left anterior descending (LAD) coronary artery was proximally ligated with a 8-0 silk suture *via* a left thoracotomy incision. After 10 min, the animals were randomized to the groups and received 30 µL intramyocardial injections of one of the following: basal medium without hEPCs (Con group) or containing 1×10^6^ non-PC hEPCs (EPCs group), BK PC hEPCs (BK PC group), BK PC hEPCs pretreated with HOE140 (BK PC/HOE group) and LY294002 (BK PC/LY group) and L-NAME (BK PC/LN group). The injections were performed at multiple sites (average of 3 to 4 sites/animal) in the free wall of the left ventricle (LV) under direct vision. After the chests of the animals were sutured, the animals were allowed to recover. A total of 112 nude mice were used in this experiment. During the operation, 15 mice died of bleeding and malignant arrhythmia, whereas, 13 mice died of infection after the operation. This experiment was divided into two subgroups, day 2 group (n = 50) and day 10 group (n = 62). Each subgroup had seven groups; 5 to 6 live nude mice were used in each group.

Prior to heart transplantation, a cell suspension containing 1×10^6^ hEPCs was labeled with carbocyanine near-infrared dye 1, 1′-dioctadecyl-3,3,3′,3′- tetramethylindodicarbocyanine,4-chlorobenzenesulfonate salt (DiD; Invitrogen, Carlsbad, CA, USA) according to the manufacturer’s instructions. 

### Echocardiographic Analysis and Heart/Body Weight Measurement

Cardiac function was evaluated at a baseline examination prior to the operation, 10 days after MI, using transthoracic echocardiography prior to sacrifice (Vevo 770TM; Visual Sonic, Toronto, Canada). Left parasternal short-axis two-dimensional M-mode images at the level of papillary muscles were recorded using a 30-MHz linear transducer. Left ventricular end-diastolic volume (LVEDV), left ventricular end-systolic volume (LVESV), left ventricular internal diameter at end-diastole (LVIDd), and left ventricular internal diameter at end-systole (LVIDs) were measured at the anterior wall, from the short-axis view, just below the level of the papillary muscle. The left ventricular ejection fraction (LVEF) and left ventricular fractional shortening (LVFS) were calculated using standard M-mode echocardiographic equations (EF = (LVEDV – LVESV) × 100%/LVEDV; FS = (LVIDd –LVIDs) × 100%/LVIDd). All measurements were averaged for five consecutive cardiac cycles and performed by an experienced examiner in a blinded fashion. 

After determining cardiac function using echocardiography, the heart was perfused with PBS and rapidly excised. After drying using a filter paper, the heart was weighed using an electronic balance. The heart weight/body weight index was calculated as heart weight/body weight ×100.

### Histological Analysis

At the end of the procedure, cardiac tissues were fixed in 4% paraformaldehyde and embedded in paraffin. To measure infarct size after myocardial infarction, we sectioned the tissue transversely in the middle of LV containing the infarcted area and subjected this section to Masson’s trichrome staining using a staining kit (Sigma) according to the instructions of the manufacturer. The infarct area was distinguished by Masson staining using computer-assisted planimetry and was expressed as the percentage of scare to total LV circumference as previously described [18]. 

DNA fragmentation was determined *via* terminal deoxynucleotidyl transferase-mediated dUTP nick end labeling (TUNEL) assay using 4-μm thick paraffin embedded sections. The procedure was performed using an *in situ* cell death detection kit (Fluorescein, Roche, Mannheim, Germany) according to the manufacturer's instructions. TUNEL-positive cardiomyocytes in the ischemic myocardium were carefully distinguished from TUNEL-positive non-cardiomyocytes by observing the morphology of each cell *via* phase contrast microscopy. An experienced investigator blinded to the treatment groups evaluated all sections. The data was expressed as the ratio of TUNEL-positive cardiomyocytes to the total number of cardiomyocytes.

### Optical imaging (OI) Studies

OI experiments were performed using a CRi Maestro *in vivo* molecular imaging system (CRi, Woburn, MA, USA), which covers the red, far-red, and near infrared (NIR) spectral regions. Light and cube images were obtained for *ex vivo* study. The recipient nude mice were anesthetized *via* isoflurane inhalation (1.5%) and placed in the imaging chamber. *Ex vivo* imaging of explanted organs (heart, lung, liver, spleen, and kidney) was performed for each animal at 2 or 10 days after injection. NIR fluorescent signal average intensities from explanted organs were quantified as counts per second per pixels using the software from CRi Maestro. The excitation wavelength coverage was set at 595 nm to 800 nm, and the emission coverage was set at 660nm to 680 nm.

### Immunofluorescence Colocalization Studies and Measurement of Myocardial Ttissues Caspase-3 Activity

The transplanted animals were sacrificed 2 or 10 days following transplantation. Ex vivo imaging was conducted immediately afterwards. Tissues were frozen in optimum cutting temperature compound (OCT compound, Miles Laboratories, Naperville, IL, USA) and sectioned into 5-µm samples using a cryostat (LeiCa CM1950, Nussloch, Germany). The cryostat sections of the left ventricular from days 2 and 10 groups were fixed with acetone for 20 min, washed in PBS, and incubated with TUNEL reaction mixture for 1 h at room temperature. All sections were counterstained with 4′,6-diamidino-2-phenylindole (DAPI) (Sigma). The multiple immunofluorescence- conjugated specimens were evaluated using a confocal microscope (FV-1000, Olympus, Tokyo, Japan).

Myocardial tissues caspase-3 activity was also measured using a caspase-3 colorimetric assay kit (KeyGEN Biology Inc.), according to the instructions of the manufacturer. In brief, myocardial tissue (100 mg) was homogenized in 50 μl lysis buffer followed by centrifugation (16,000 × g, 4°C, 20 minutes). Following procedures were almost identical to the cell caspase-3 activity detection.

### Statistical Analysis

Data were expressed as mean ± SEM. The data of the experimental groups were compared using one-way ANOVA followed by Fisher’s PLSD. Differences were considered statistically significant at a value of *P* < 0.05.

## Results

### Characterization of hEPCs

**Figure 1:**
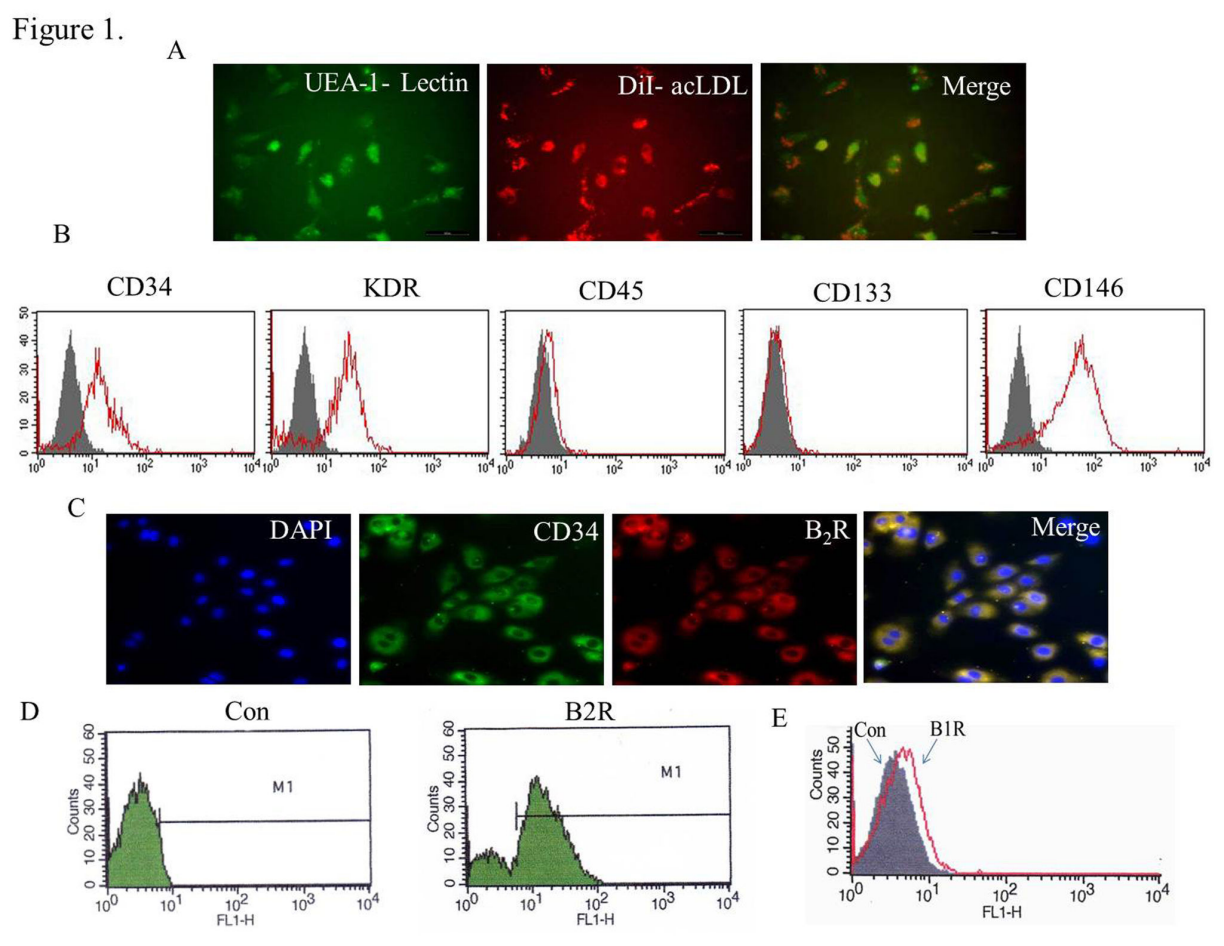
Characterization of cultured hEPCs and the expressions of B1R and B2R in hEPCs. (A) At day 7 following isolation, the adherent cells intensively took up acLDL and bound an endothelial-specific lectin, as assessed using fluorescence microscopy. (Original magnification: 400×) (B) Representative flow cytometric analyses of hEPCs for expression of the cell surface markers. The hEPCs of passage 3 were positive for CD34, KDR, and CD146, whereas negative for CD45 and CD133. Filled areas show isotype control staining, empty areas represent specific marker expression. (C) Representative immunofluorescence staining of hEPCs for co-expression of B2R with the progenitor lineage marker CD34. (Original magnification: 400×) (D and E) B1R and B2R expressions in hEPCs were detected *via* flow cytometry.

The mononuclear cells derived from human umbilical cord blood were separated using density gradient centrifugation and differentiated into “late-outgrowth EPCs” after a long culture period (three to six passages). The endothelial cell phenotype was characterized by assessing the acLDL-DiI uptake and FITC- conjugated UEA-1-lectin binding (Figure 1A). The hEPCs were positive for CD34, KDR, and CD146, whereas negative for CD45 and CD133 (Figure 1B). The cells were therefore confirmed as hEPCs. In addition, hEPCs expressed high levels of B2R and co-expressed B2R, as well as the EPC marker CD34 (Figures 1C and D). The B1R expression in hEPCs was rarely detectable (Figure 1E).

### Cytoprotective Effect of Appropriate Concentrations and Time Points of BK PC on Cell Apoptosis

**Figure 2:**
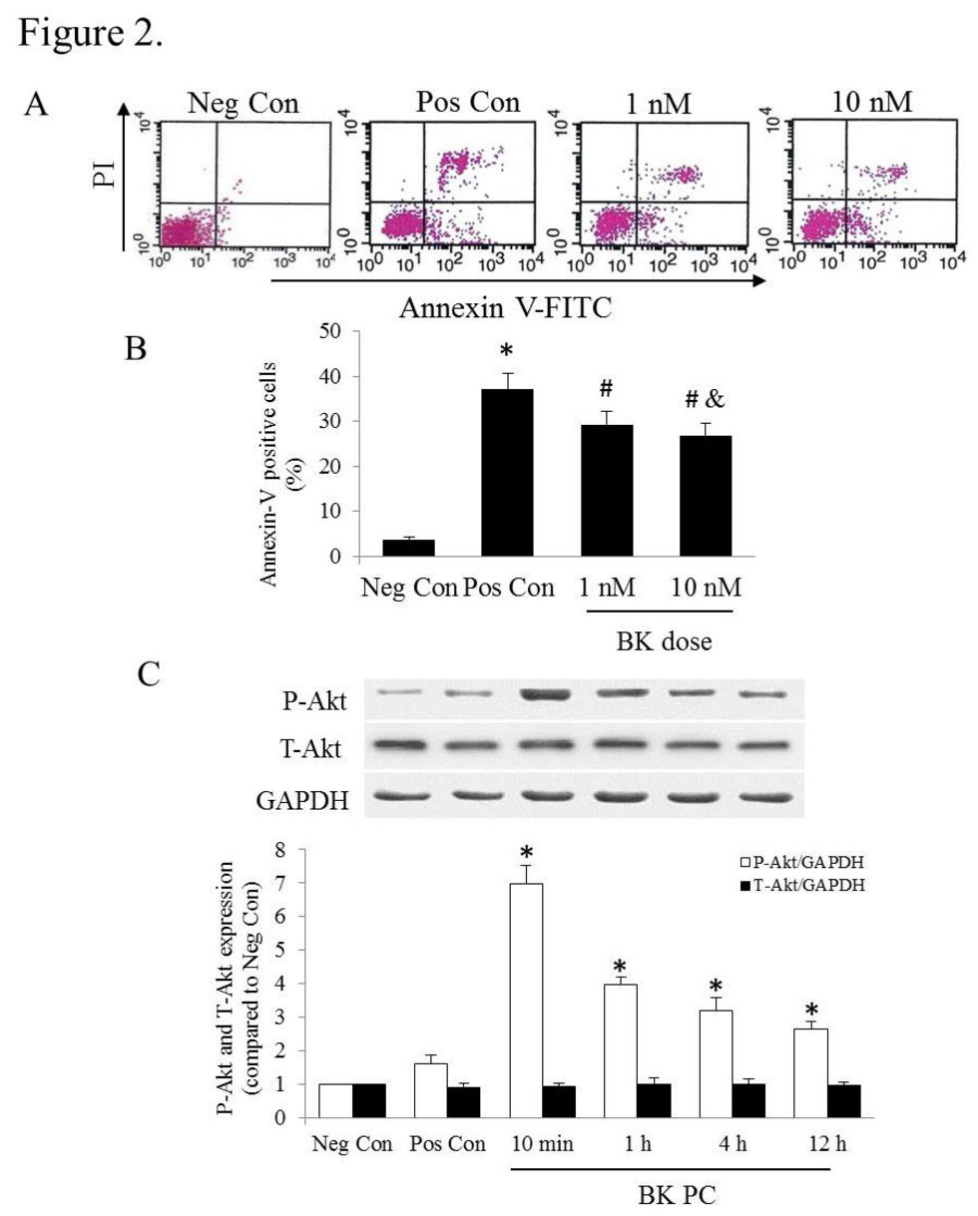
Effects of various BK concentrations and BK preconditioning time points on apoptosis. (A) The hEPCs were cultured with various concentrations of BK PC (1 and 10 nM) for 10 min with RPMI-1640 medium, and then apoptosis was induced by hypoxia along with serum deprivation for 12 h. Representative flow cytometry analysis of apoptotic cells after being labeled with annexin V and propidium iodide. The apoptotic cells were positively stained with annexin V. (B) Quantitative analysis of apoptotic cells (annexin V positive). Values are expressed as mean ± SEM. n = 6 for each group. *P < 0.01 vs. Neg Con group; ^#^ *P* < 0.01 vs. Pos Con group; ^&^ *P* > 0.05 vs. 1 nM of BK preconditioning. (C) Time course of Akt activation by BK preconditioning, as determined using Western blots for phosphor-Ser^473^-Akt and total Akt, which were normalized to GAPDH. Representative blots are shown in the upper panel and densitometric quantitation of protein expression levels are shown as fold changes in the lower panel. Values are expressed as mean ± SEM. n = 5 for each group; *P < 0.01 vs. corresponding Pos Con group.

The annexin-V positive cell rate of 1 and 10 nM BK preconditioned hEPCs was significantly lower compared with non-preconditioned hEPCs (*P* < 0.01), as shown in Figure 2A and B. Although the number of apoptotic cells in the 10 nM BK group was fewer than that in the 1 nM BK group, the difference between the two concentrations was not statistical significance. The time course of Akt activation by 10 nM BK PC based on the Western blots are shown in Figures 2C. BK PC induced a significantly higher in phospho-Akt by 10 min (*P* < 0.01 vs. Pos Con). The phospho-Akt expression was sustained for 12 h, which was significantly higher than normal level. Based on these results, we selected the PC of hEPCs at 10 min with 10 nM BK for our subsequent study.

### Cytoprotective Effects of PC on hEPC Apoptosis

**Figure 3:**
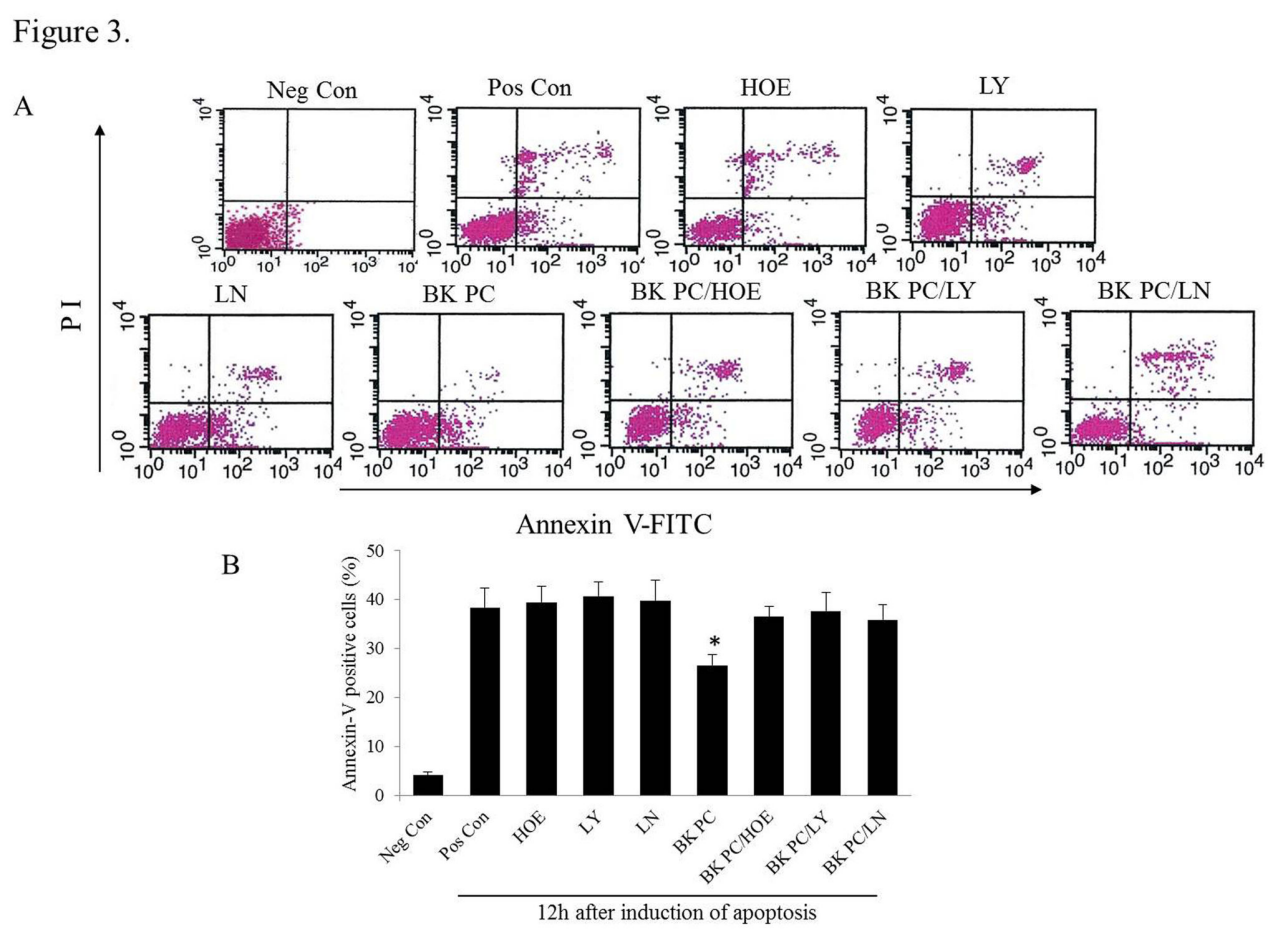
Inhibitory effect of BK preconditioning on hypoxia along with serum deprivation-induced apoptosis of hEPCs. (A) hEPCs were exposed to hypoxia along with serum deprivation for 12 hours. Representative flow cytometry analysis of apoptotic cells after being labeled with annexin V and propidium iodide. (B) Quantitative analysis of the apoptotic cells (annexin V positive). Data were obtained from six independent experiments and are expressed as mean ± SEM. *P < 0.01 *vs*. other groups during cell apoptosis.

The annexin-V positive cells detected using flow cytometry in the BK PC group were significantly lower compared with the Pos Con group (*P* < 0.01). The anti-apoptotic effect of BK PC was abolished by HOE140, LY294002 and L-NAME (*P* < 0.01; Figures 3A and B). Moreover, although the number of apoptotic cells in the HOE group, LY group and LN group was more than that in the Pos Con group, the difference among these three groups was not statistical significance. These findings suggest that the hEPCs apoptosis induced by serum deprivation is inhibited by BK PC and partly dependent on the B2R-dependent Akt/eNOS pathway. 

### PC Activates the B2R-dependent Akt/eNOS Pathway

**Figure 4:**
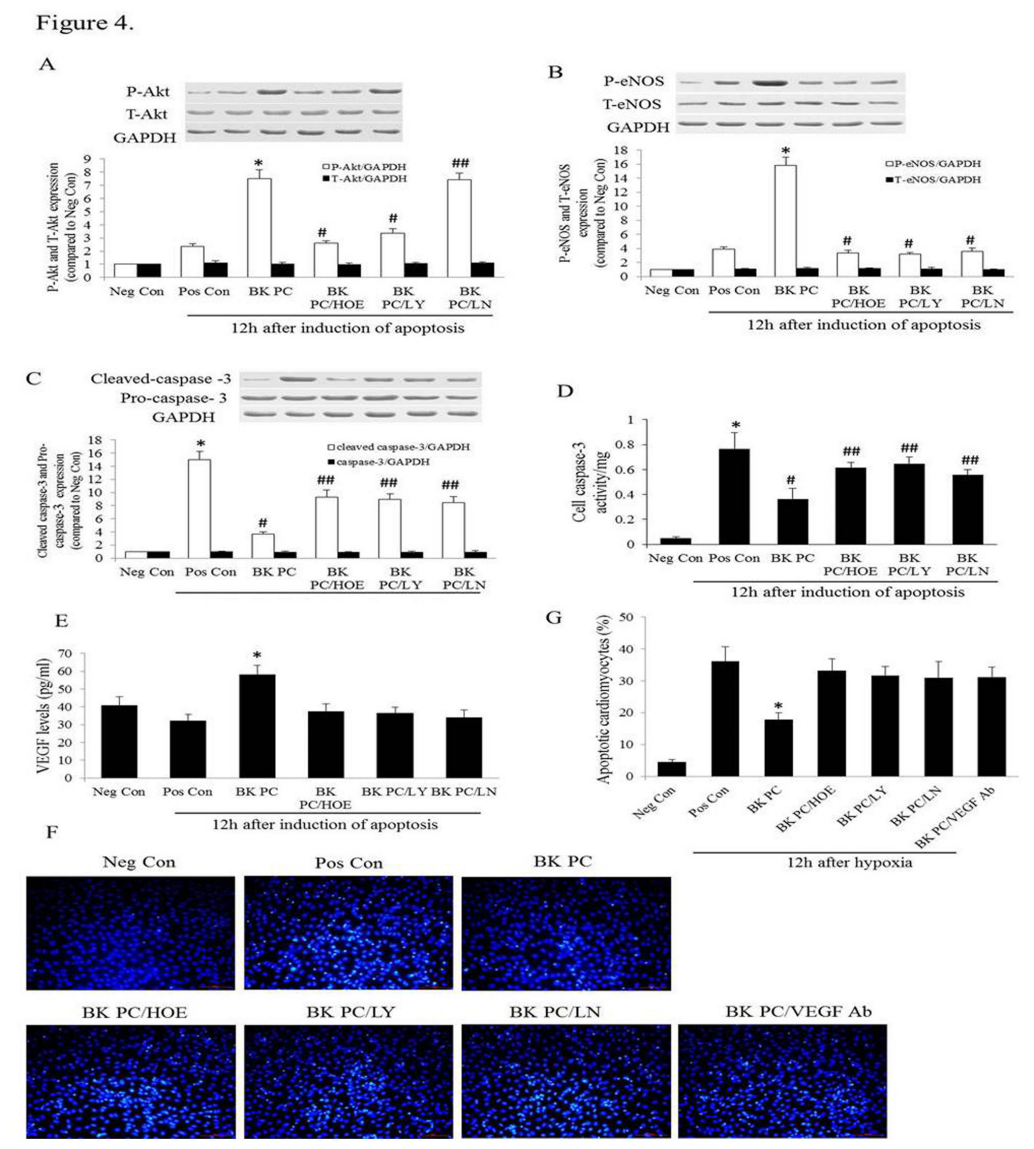
The mechanism of BK preconditioning on the anti-apoptosis effect of hEPCs. (A and B) Western blots analysis to see the expression of P-Akt, T-Akt, P-eNOS and T-eNOS at 12 hours after apoptosis induction. Representative blots are shown in the upper panel and densitometric quantitation of protein expression levels are shown as fold changes in the lower panel. Values are expressed as mean ± SEM. n = 5 for each group; *P < 0.01 vs. corresponding Pos Con group, ^#^ *P* < 0.01 *vs*. corresponding BK PC group, ^##^ *P* > 0.05 *vs*. corresponding BK PC group. (C) Western blots analysis to see the expression of Cleaved-caspase-3 and Pro-caspase-3 at 12 hours after apoptosis induction. Representative blots are shown in the upper panel and densitometric quantitation of protein expression levels are shown as fold changes in the lower panel. Values are expressed as mean ± SEM. n = 5 for each group; *P < 0.01 vs. corresponding Neg Con group, ^#^ *P* < 0.01 *vs*. corresponding Pos Con group, ^##^ *P* < 0.05 *vs*. corresponding BK PC group. (D) Cell caspase-3 activity in different groups undergoing hypoxia along with serum deprivation–induced apoptosis for 12 hours. Values are expressed as mean ± SEM. n = 6 for each group; *P < 0.01 vs. Neg Con group, ^#^ *P* < 0.01 *vs*. Pos Con group, ^##^ *P* < 0.05 *vs*. BK PC group. (E) VEGF levels in the cell supernatant of various treatment groups under hypoxia along with serum deprivation. After 12 hours of incubation, conditioned medium from control and treated cells (n = 3) was subjected to VEGF ELISA assay. VEGF concentration values are mean ± SEM. *P < 0.01 vs. other groups during cell apoptosis. ELISA data are representative of three independent experiments. (F) Effects of the hEPCs conditioned medium on apoptosis of neonatal rat ventricular cardiomyocytes exposed to hypoxia. Representative Hoechst 33342 staining images (left panel) in neonatal rat ventricular cardiomyocytes treated with hypoxia for 12 hours. Hoechst 33342 staining for nuclear morphology was performed to assess apoptotic cell death (Original magnification: 200×; Scale bar is 200 px). (G) Quantitative analysis of apoptosis levels using Hoechst 33342 staining in various conditioned medium 12 hours after hypoxia. Values are expressed as mean ± SEM. n = 4 for each group; *P < 0.01 vs. other groups during cell apoptosis.

As shown in Figures 4A and B, the anti-apoptotic effect of BK PC on the hypoxia along with serum deprivation-induced apoptosis of hEPCs was associated with increased Akt and eNOS phosphorylation, which was abolished by their antagonists, LY294002 and L-NAME, respectively. It was aimed to determine whether the B2R pathway was involved in BK PC mediated Akt and eNOS activation and found that the BK PC-induced Akt and eNOS phosphorylation were inhibited by HOE140 at 12 h after the apoptosis induced by serum deprivation. Moreover, the cleaved caspase-3 levels significantly decreased in the BK PC group compared with that of the other groups during apoptosis (Figure 4C). These results indicate that the anti-apoptotic effect of BK PC on hEPCs is mediated through the B2R-dependent Akt/eNOS signaling pathway. 

### BK PC Inhibits Caspase-3 Activity and Enhances VEGF Levels

Figure 4D illustrates that the hEPCs, when subjected to hypoxia and serum deprivation, showed increases in caspase-3 activity, which was significantly suppressed by BK PC (*P* < 0.01). In addition, the effect of BK PC was significantly abolished by HOE140, LY294002 and L-NAME (*P* < 0.05).

 The VEGF levels in the supernatant increased in the BK PC group compared with the Pos Con group (*P* < 0.01). However, the addition of HOE140, LY294002 or L-NAME to the culture medium blocked these effects (*P* < 0.01; Figure 4E).

### Effects of the hEPCs Conditioned Medium on Hypoxia-induced Neonatal Cardiomyocyte Apoptosis

We hypothesized that the BK PC hEPCs might release cyto-protective factors that prevent apoptosis in cardiomyocytes. To test this, neonatal rat ventricular cardiomyocytes were exposed to hypoxia for 12 h in the presence of the conditioned medium. Our results showed that exposing neonatal rat ventricular cardiomyocytes to hypoxia-conditioned medium for 12h from cultured BK PC hEPCs results in a significant reduction in the number of apoptotic cardiomyocytes compared with similarly treated medium from cultured hEPCs (17.8 ± 2.2% *vs* 36.2 ± 4.5%, *P* < 0.01). However, the number of apoptotic cardiomyocytes was increased when incubation in hypoxia-conditioned medium from cultured with HOE140, LY294002 or L-NAME prior to BK PC hEPCs, as compared with that cultured with BK PC hEPCs (*P* < 0.01). Moreover, the anti-apoptotic effect of cardiomyocytes incubated in conditioned medium of BK-PC-hEPCs was significantly reduced by the addition of neutralizing antibody against VEGF (Figures 4F and 4G).

### Effects of BK Preconditioned hEPCs Transplantation on Cardiac Function and Infarct Size after MI

**Figure 5:**
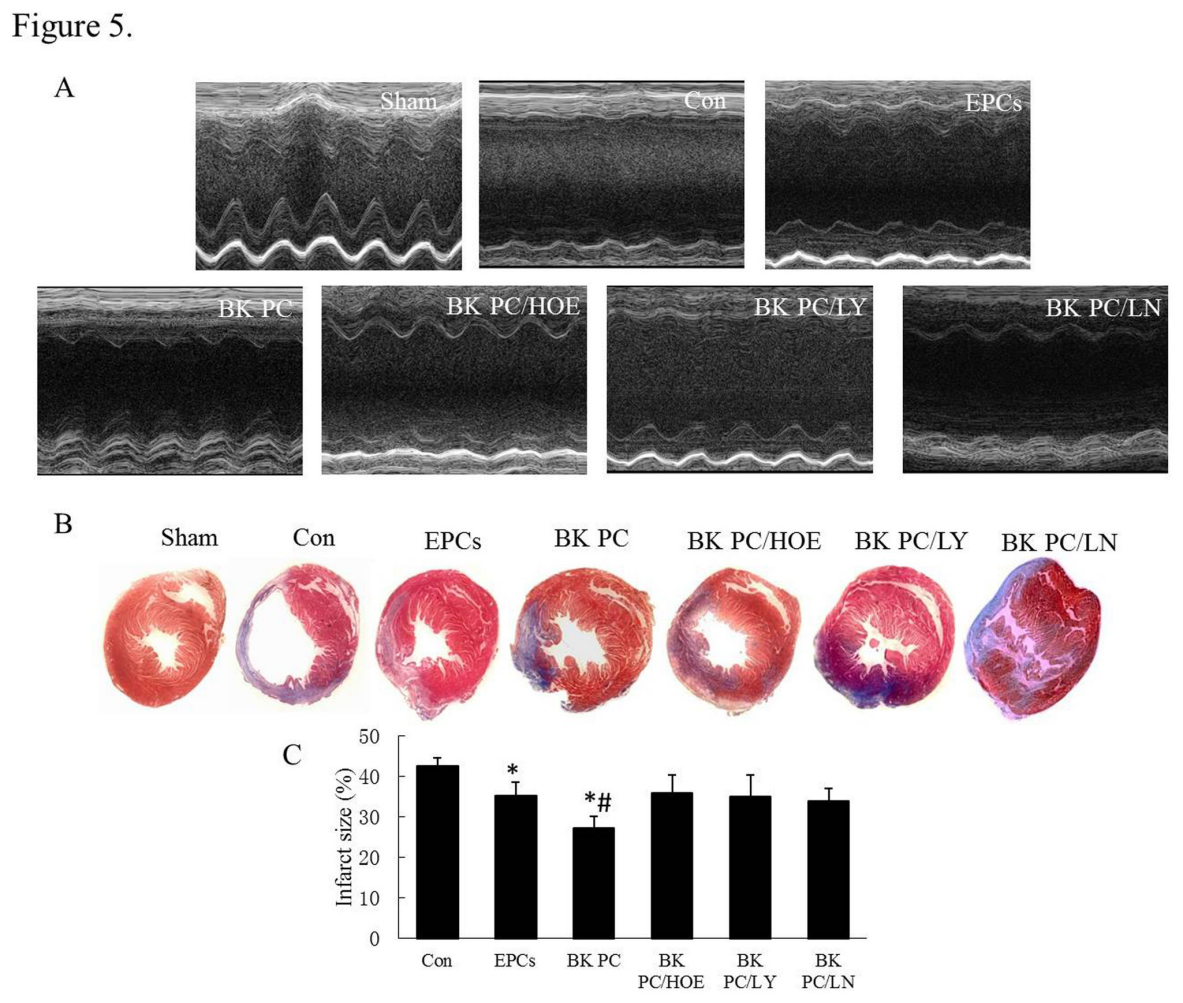
Cardiac function and infarct size measurements. (A) Echocardiographic measurements were performed 10 days after hEPCs transplantation. Representative M-mode images at the papillary muscle level were recorded for various groups. (B) Representative Masson’s trichrome-stained histological sections to measure infarct size 10 days after coronary ligation and hEPCs administration. Collagen is blue but myocardium appears red. Infarct size was calculated as the area occupied by collagen. (C) Quantitative analysis of infarct size. Values are expressed as mean ± SEM. n = 5 - 6 for each group; *P < 0.01 vs. Con group, ^#^ *P* < 0.01 *vs*. EPCs group & BK PC/HOE group & BK PC/LY group & BK PC/LN group .

Cardiac function was evaluated at 10 d following cell delivery using echocardiography (Figure 5A and Table 1). The LVIDd and LVIDs in the BK PC group were significantly lower than that in the EPCs group (*P* < 0.01). Both groups had significantly lower LVIDd and LVIDs than that in the Con group (*P* < 0.01). However, no significant differences in LVIDd and LVIDs were noted in the EPCs, BK PC/HOE, BK PC/LY and BK PC/LN groups. Furthermore, LVEF and FS were significantly improved in the BK PC group compared with that in the Con and EPCs groups, respectively (*P* < 0.01). Interestingly, the effect of PC on cardiac function was antagonized by HOE140, LY294002 and L-NAME, implicating a role for B2R and Akt/eNOS in mediating cardiac protection by BK PC.

**Table 1:** Effects of BK preconditioning on physiological parameters, cardiac function and infarct size at 10 days following myocardial infarction.

Variable	Baseline	Sham	Con	EPCs	BK PC	BK PC/HOE	BK PC/LY	BK PC/LN
HW/BW (mg/g)	5.54±0.76	5.93±0.54	7.94±0.33	7.14±0.41*	6.63±0.39 *#	7.15±0.46	7.17±0.35	7.18±0.52
LVEF(%)	83.76±1.32	81.51±0.82	28.02±2.10	35.08±2.76*	44.85±4.17*#	34.63±1.53	34.15±1.63	35.19±3.16
LVFS(%)	52.18±1.24	49.09±0.13	12.85±0.95	16.41±1.54*	26.60±2.62*#	16.73±0.71	16.31±0.70	16.97±1.15
LVIDd (mm)	3.03±0.21	3.21±0.09	5.08±0.23	4.40±0.27*	3.54±0.32*#	4.48±0.22	4.59±0.21	4.51±0.32
LVIDs (mm)	1.52±0.09	1.63±0.06	4.42±0.17	3.68±0.26*	2.60±0.25*#	3.73±0.22	3.80±0.21	3.67±0.51
Infarct size (%)	0	0	42.55±1.93	35.27±3.23*	27.28±2.75*#	35.76±4.52	35.04±5.25	35.06±4.97

Values are expressed as mean ± SEM. MI mice were randomized to one of six experimental groups.

HW, heart weight; BW, body weight. LVEF, left ventricular ejection fraction; LVFS, left ventricular fractional shortening; LVIDd, left ventricular internal diameter at end-diastole; LVIDs, left ventricular internal diameter at end-systole. Baseline, at the beginning of the normal model construction prior to the operation in the studied mice; BK, bradykinin; PC, preconditioning; HOE, HOE140; LY, LY294002; LN, L-NAME. ^*^ *P* < 0.01 *vs.* Baseline, Sham, and Con group, ***^#^*** *P* < 0.01 *vs.* EPCs group. n = 5 - 6 for each group.

Based on the Masson’s trichrome staining and quantitative analysis (Figures 5B + C and Table 1), the BK PC group had a remarkably reduced infarct size in the left ventricle compared with the Con and EPCs groups (*P* < 0.01) at 10 d following hEPCs transplantation. The effect of BK PC on MI was also abolished by HOE140, LY294002 and L-NAME (*P* < 0.01). In addition, the heart weight/body weight ratio in the BK PC group was substantially lower than that in the other MI groups (Table 1).

### Effects of BK PC on Transplanted Cells Survival and Cardiomyocytes Apoptosis in the Ischemic Heart

**Figure 6:**
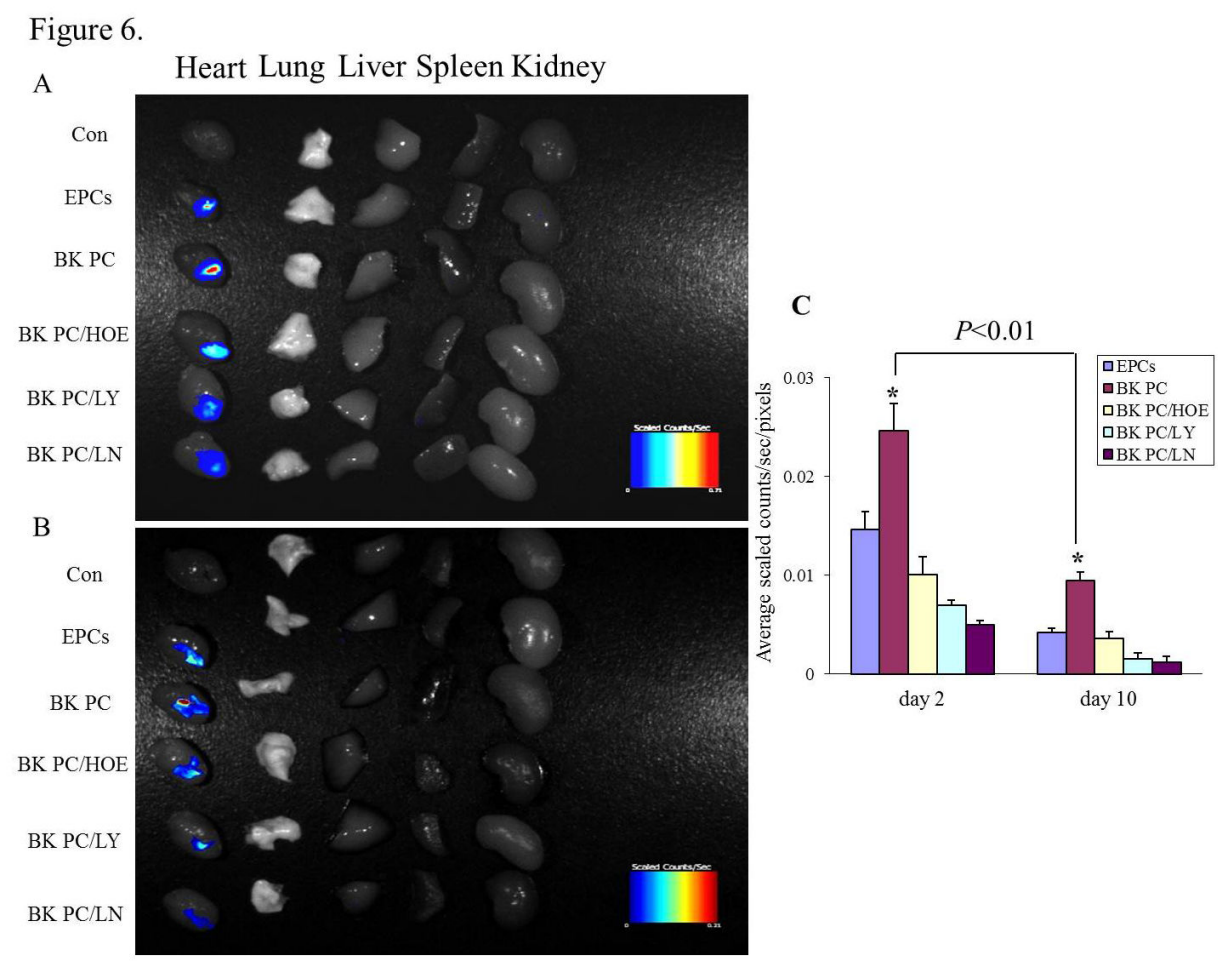
Ex vivo optical imaging study. (A and B) Representative NIR fluorescent images of explanted organs at 2 days or 10 days following the implantation of DiD-labeled hEPCs into the ischemic myocardium of nude mice. Bars represent maximum radiance. (C) Quantitative analysis of NIR fluorescent signals in the explanted hearts of each group at two time points. (A: 2 days after cell delivery, B: 10 days after cell delivery). All values are expressed as mean ± SEM. n = 5 for each group, *P < 0.01 *vs*. other myocardial infarction groups.

The *ex vivo* NIR fluorescent signals of the transplanted DiD-labeled hEPCs in nude mice hearts were evaluated in all groups using OI (Figures 6A to C). Most of the fluorescent signals were observed in the heart compared with that in the other organs (lung, liver, spleen, and kidney) of all groups at days 2 and 10. Moreover, the fluorescent signals of the heart in the BK PC group were remarkably higher than that in the other groups at two time points. The fluorescent signals in the heart of the BK PC group were 1.68 to 2.26 fold higher than that in the EPCs group at two time points (0.0246 ± 0.0018 vs. 0.0146 ± 0.0028; 0.0095 ± 0.0008 vs. 0.0042 ± 0.0004, respectively, *P* < 0.01). The fluorescent signals of the explanted hearts in each group gradually decreased from day 2 to day 10.

**Figure 7:**
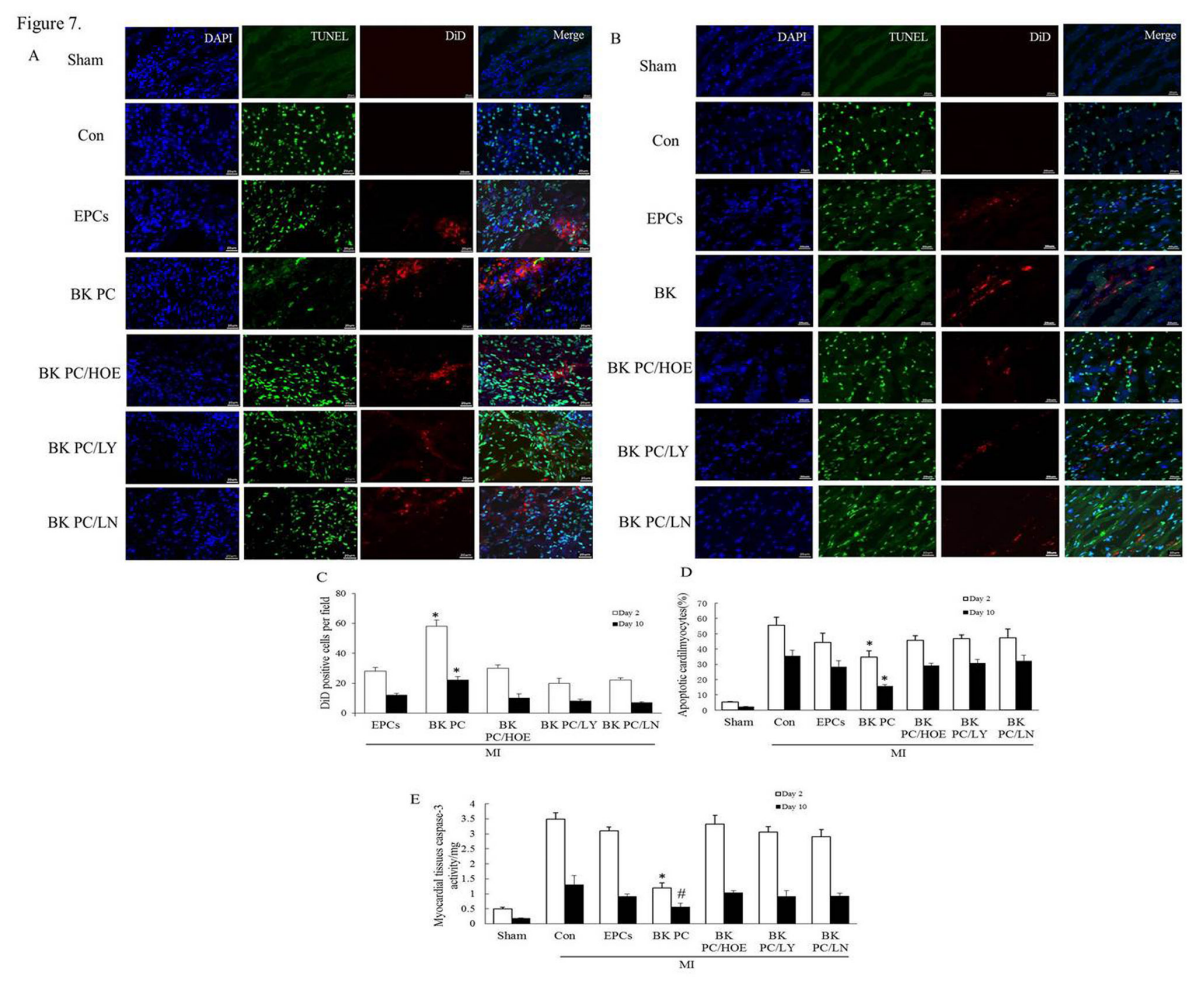
Effects of BK preconditioning on cardiomyocytes apoptosis and measurement of caspase-3 activity in the myocardium. (A and B) TUNEL-positive cardiomyocytes (green) and NIR fluorescent signals (red) were both observed in the myocardium at days 2 and 10. Counterstaining was performed using DAPI (blue). TUNEL-positive non- cardiomyocytes were excluded. Original magnification: 800×. Scale bar = 20 μm. (A: 2 days after cell delivery, B: 10 days after cell delivery). (C) Quantitative analysis of transplanted hEPCs at days 2 and 10 after cell delivery. DAPI- (blue) and DiD- (red) positive cells were counted using Image-Pro Plus 6.0 software. Double-labeled cells in each field were counted as surviving hEPCs. n = 5 for each group. (D) Quantitative analysis of apoptotic cardiomyocytes expressed as percentage of TUNEL-positive nuclei in cardiomyocytes at days 2 and 10 after cell delivery. n = 5 for each group. (E) Cardiac tissue caspase-3 activity of different groups at days 2 and 10 following cell delivery. n = 6 for each group. All values are expressed as mean ± SEM. *P < 0.01, ^#^ *P* < 0.05 *vs*. other corresponding myocardial infarction groups.

The BK PC group had more DiD-labeled transplanted cells and less TUNEL-positive cardiomyocytes than that of the other groups at days 2 and 10 based on confocal microscopy, as shown in Figures 7A and B. The quantity of transplanted hEPCs double-labeled with DiD and DAPI within the cardiac tissues in the BK PC group were significantly greater than those in the Con and EPCs groups 2 and 10 d following transplantation. The numbers of DiD labeled hEPCs were decreased in the BK PC/HOE, BK PC/LY and BK PC/LN groups as compared with those in the BK PC group (Figure 7C, P < 0.01). Moreover, the ratio of TUNEL-positive cardiomyocytes to total number of cardiomyocytes in the BK PC group was significantly reduced as compared to the Con and EPCs groups at day 2 and 10 after cell delivery (Figure 7D). However, the protective effect of BK PC was blocked by HOE140, LY294002 and L-NAME (*P* < 0.01).

The caspase-3 activity in the myocardium was inhibited in the BK PC group compared with that in the Con and EPCs groups at days 2 and 10 after cell delivery (*P* < 0.05). This effect was abolished by HOE140, LY294002 and L-NAME (*P* < 0.05; Figure 7E). These results indicate that the therapeutic effects of hEPCs transplantation in a healing MI were enhanced by BK PC via B2R-dependent Akt/eNOS signaling pathway. 

## Discussion

This study is the first to demonstrate that BK PC enhances survival and anti- apoptotic effect of hEPCs, thereby improving cardiac function following transplantation in the ischemic myocardium. The major findings are as follows: 1) BK PC protected hEPCs against apoptosis through a mechanism that is dependent, at least in part, on the B2R dependent PI3K/Akt/eNOS signaling pathway. 2) PC hEPCs with BK reduced the infarct size and improved cardiac performance at 10 days following MI by increasing transplanted cell survival, decreasing cell apoptosis. 

IPC is a very powerful protective phenomenon against lethal ischemic injury. BK has been considered as one of the mediators for early and delayed IPC [19]. Although previous studies have demonstrated that BK pretreatment could improve post-ischemic cardiac function in both experimental studies [20] and clinical trials [21], the positive effects of PC stem cells with BK prior to transplantation are still uncertain. In this study, we applied pharmacologic PC to hEPCs with BK and found that BK PC protects hEPC survival and mitigates cell apoptosis. 

The PI3K/Akt signaling pathway has a crucial function in limiting apoptosis [22]. Our present study shows that the effect of BK PC on hEPCs apoptosis was blocked by LY294002, suggesting that BK PC protects the hEPCs against apoptosis through the PI3K/Akt signaling pathway. This result is in agreement with a previous study [23]. However, we also observed that the Akt phosphorylation in BK PC hEPCs was not inhibited by L-NAME, indicating that eNOS is an important downstream target of Akt [23,24]. BK has been found to induce the phosphorylation and activation of eNOS *via* Akt [20,23]. Our data shows that BK PC promoted eNOS phosphorylation, which was blocked by LY294002. These findings suggest that the activated eNOS is PI3K/Akt-dependent and play an important role in mediating protection against hEPCs apoptosis. Alternatively, caspase-3 is one of the key players in apoptosis [25] and a well-identified downstream target for PI3K/Akt/eNOS. BK PC induced the inactivation of terminal caspase-3, which may lead to the inhibition of myocardial apoptosis following cardioplegic I/R [14]. In the current study, the cleaved caspase-3 levels were increased in hEPCs following serum deprivation- induced apoptosis and the caspase-3activation was suppressed by BK PC. Moreover, our present study also showed that this Akt/eNOS/caspase-3 pathway was inhibited by HOE140, indicating that BK exerts its effect on hEPCs *via* B2R, which is consistent with a previous study [12]. Thus, our results validate that BK PC exerts its anti-apoptotic effect on hEPCs through a B2R dependent Akt /eNOS/caspase-3 signal pathway.

Our data demonstrated the cytoprotective effect of hypoxic conditioned medium from cultured BK PC-hEPCs on isolated neonatal rat ventricular cardiomyocytes exposed to hypoxia *in vitro*. Moreover, *In vivo* data also confirmed that transplantation of BK PC hEPCs could prevent cardiomyocytes apoptosis in the ischemic heart (see Figure S1). The effect of inhibition of cardiomyocyte apoptosis may be associated with the cyto-protective factors released by BK PC- hEPCs. *In vitro* data demonstrated a significant increase of VEGF secretion by BK PC-hEPCs. VEGF is well known as a proangiogenic cytokine, which can be secreted by stem cells [26]. However, our results agree with the earlier findings that apart from stimulating angiogenesis capacities, VEGF itself could also inhibit cardiomyocyte apoptosis [27]. 

In the present study, we used the NIR dye DiD to label the transplanted cells. DiD has low cytotoxicity and high resistance to intercellular transfer and autofluorescence [28]. Furthermore, based on OI for *in vitro* cell labeling and *in vivo* cell tracking [28], we observed that hEPCs could be efficiently labeled using DiD *in vitro*. The fluorescence counts also correlated linearly with cell number increase (see Figure S2), allowing cell engraftment to be assessed in explanted hearts. *Ex vivo* studies *via* NIR OI show that more transplanted hEPCs survived in the myocardium in the BK PC group at days 2 and 10 than that in the other groups. However, OI-based cell tracking techniques have limited penetration depth, limited quantification and poor spatial resolution because of scattering [29]. In future studies, we plan to use firefly luciferase or multimodal imaging for stem cell labeling and tracking to obtain the most effective technique possible.

In conclusion, this is the ﬁrst study to show that BK PC resists apoptosis partly through the B2R-dependent Akt/eNOS pathway. PC with BK improves cardiac performance by enhancing transplanted cell survival, preventing cell apoptosis in the hostile environment following acute MI. Thus, the BK PC of stem cells is a novel, cell-based therapeutic approach that provides novel molecular mechanisms against cell apoptosis and cardiac remodeling, and has potential clinical applications. 

## Supporting Information

Figure S1 **Effect of BK preconditioning on transplanted hEPCs survival and cardiomyocyte apoptosis in the infarcted myocardium at day 10 following cell delivery.** (A) Representative immunofiuorescent micrographs of the hearts transplanted with DiD-labeled hEPCs. Original magnification: 400×. (B) Quantitative analysis of DiD positive cells per field. (C) Representative photomicrographs of TUNEL- positive apoptotic cardiomyocytes in the left ventricular of nude mice hearts. Original magniﬁcation: 200 ×. (D) Apoptotic cardiomyocytes are expressed as a percentage of TUNEL- positive nuclei in cardiomyocytes. TUNEL-positive non-cardiomyocytes were excluded. Scale bar = 50 μm. All values are expressed as mean ± SEM. n = 5 for each group, *P < 0.01 *vs*. other myocardial infarction groups.
(TIF)

Figure S2 ***Invitro* hEPCs of the DiD-labeling and the correlations of NIR fluorescent signal and the cell number.** (A) DiD-labeled cells appeared red *via* fluorescence microscopy (Original magnification is 100×; excitation wavelength, 595–800 nm; emission wavelength, 660–680 nm). (B) Fluorescent images show that the signal intensity increased with increasing cell number. Bars represent maximum radiance. (C) Correlation plot shows fluorescence counts correlated linearly with cell number (y=0.0083x + 0.0004; r^2^ = 0.9977).
(TIF)

Results S1 **These are the results for Figure S1.**
(DOCX)

Results S2 **These are the results for Figure S2.**
(DOCX)
