# Supplementary figures and images for: Bradykinin Preconditioning Improves Therapeutic Potential of Human Endothelial Progenitor Cells in Infarcted Myocardium
Source: PLoS One. 2013 Dec 2;8(12):e81505. doi: 10.1371/journal.pone.0081505 (PMC3846887; doi:10.1371/journal.pone.0081505)

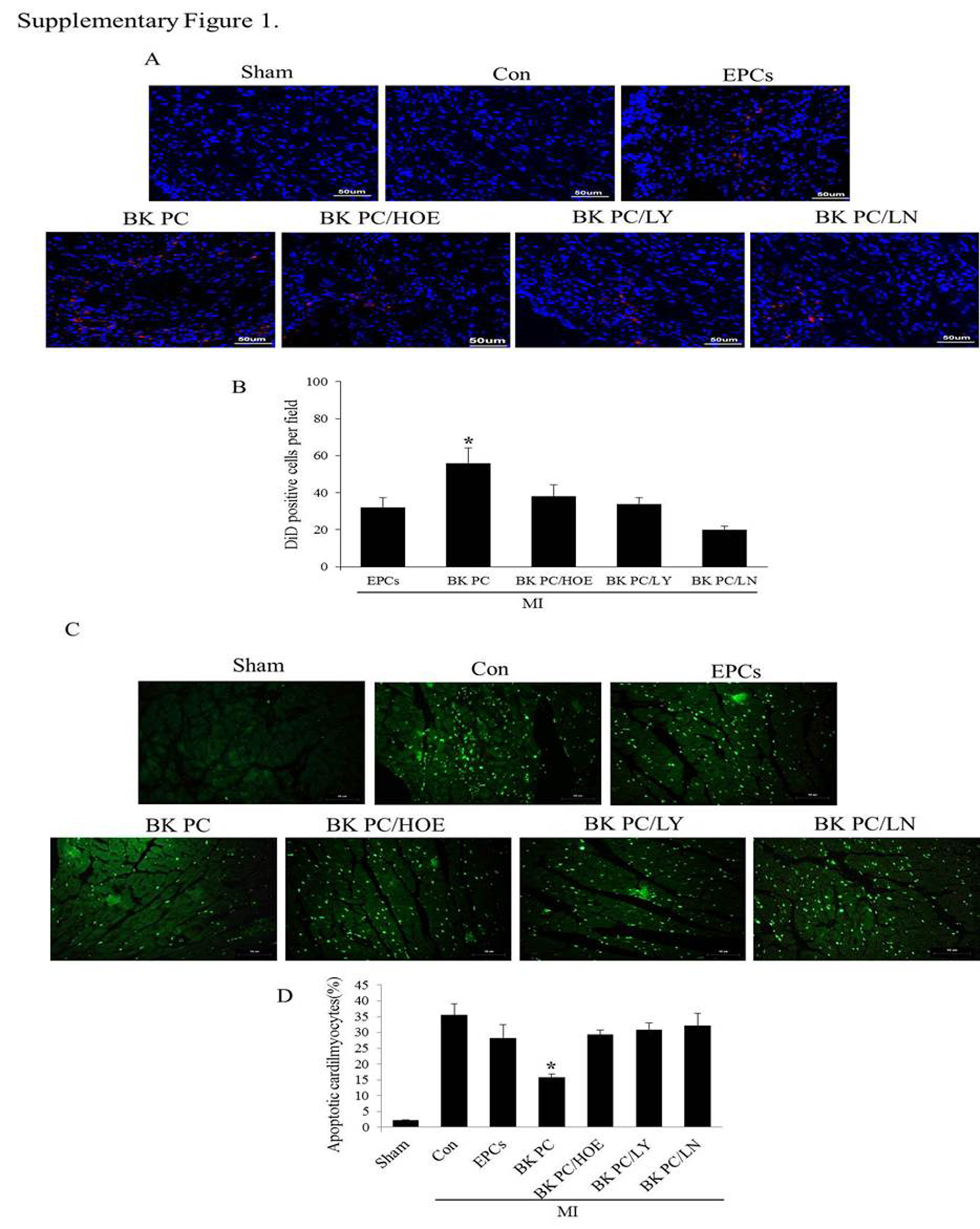

Supplement: Figure S1 — Effect of BK preconditioning on transplanted hEPCs survival and cardiomyocyte apoptosis in the infarcted myocardium at day 10 following cell delivery. (A) Representative immunofiuorescent micrographs of the hearts transplanted with DiD-labeled hEPCs. Original magnification: 400×. (B) Quantitative analysis of DiD positive cells per field. (C) Representative photomicrographs of TUNEL- positive apoptotic cardiomyocytes in the left ventricular of nude mice hearts. Original magniﬁcation: 200 ×. (D) Apoptotic cardiomyocytes are expressed as a percentage of TUNEL- positive nuclei in cardiomyocytes. TUNEL-positive non-cardiomyocytes were excluded. Scale bar = 50 μm. All values are expressed as mean ± SEM. n = 5 for each group, *P < 0.01 vs. other myocardial infarction groups. (TIF) [file pone.0081505.s001.tif]

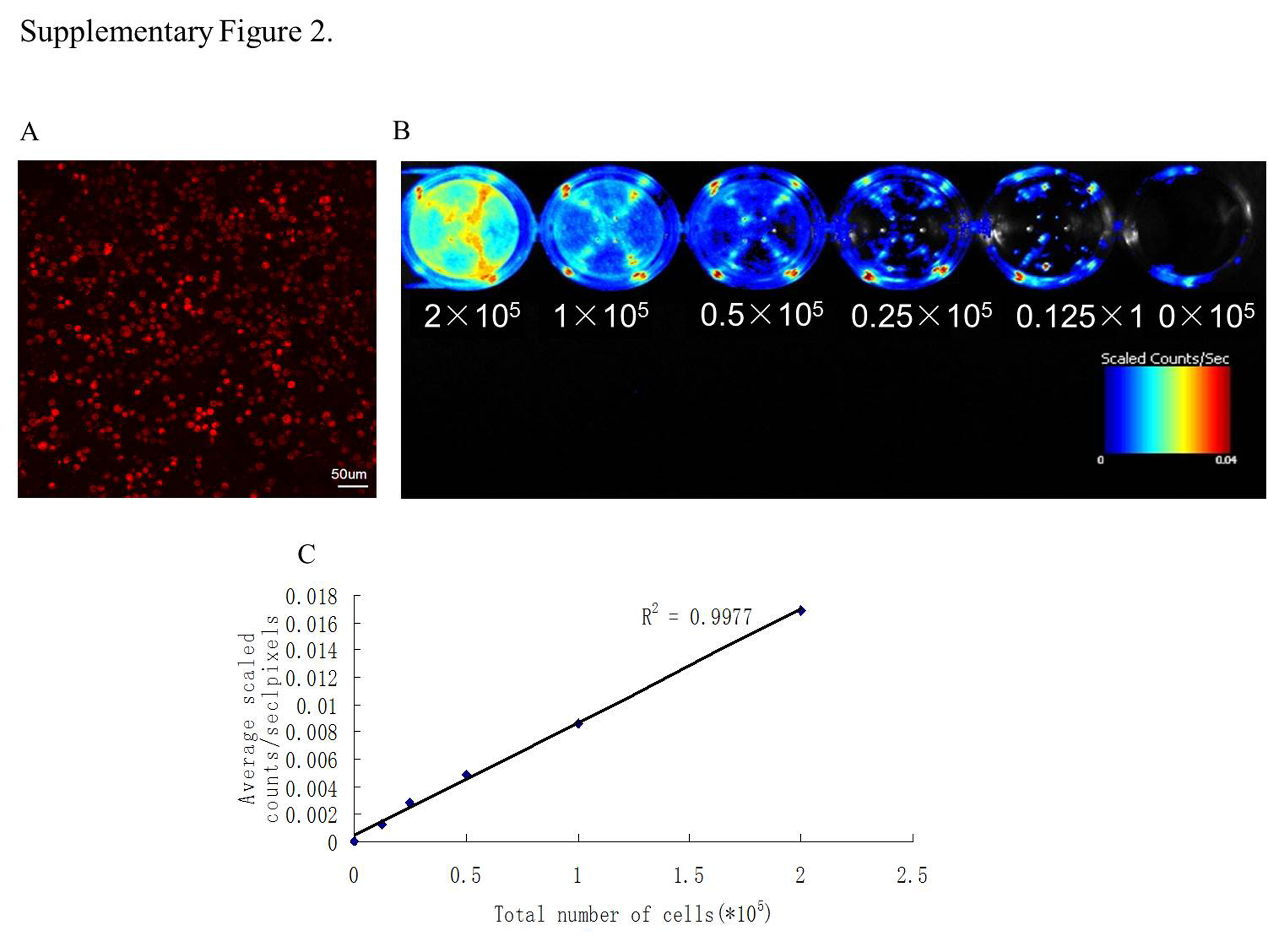

Supplement: Figure S2 — Invitro hEPCs of the DiD-labeling and the correlations of NIR fluorescent signal and the cell number. (A) DiD-labeled cells appeared red via fluorescence microscopy (Original magnification is 100×; excitation wavelength, 595–800 nm; emission wavelength, 660–680 nm). (B) Fluorescent images show that the signal intensity increased with increasing cell number. Bars represent maximum radiance. (C) Correlation plot shows fluorescence counts correlated linearly with cell number (y=0.0083x + 0.0004; r2 = 0.9977). (TIF) [file pone.0081505.s002.tif]
