# Supplementary material for: Bradykinin Preconditioning Improves Therapeutic Potential of Human Endothelial Progenitor Cells in Infarcted Myocardium
Source: PLoS One. 2013 Dec 2;8(12):e81505. doi: 10.1371/journal.pone.0081505 (PMC3846887; doi:10.1371/journal.pone.0081505)
Supplement: Results S1 — These are the results for Figure S1. (DOCX) [file pone.0081505.s003.docx]

*BK PC promotes transplanted hEPC survival and reduces cardiomyocyte apoptosis in the infarcted myocardium*

The number of transplanted hEPCs in the recipient heart was identified using DiD labeling. A significant increase in DiD-positive cells was observed at the site of the left ventricular in the BK PC group compared with that in the EPCs group (*P* < 0.01). These effects were blocked by HOE140, LY294002 and L-NAME (*P* < 0.01; Figures S1A and B). In addition, the apoptotic cardiomyocytes were detected using TUNEL staining in the infarcted myocardium at 10 d after MI (Figure S1C). The ratio of TUNEL-positive cardiomyocytes to the total number of cardiomyocytes in the BK PC group significantly decreased compared with that in the Con and EPCs groups (*P* < 0.01; Figure S1D). However, the protective effect of BK PC was also abrogated by HOE140, LY294002, and L-NAME (*P* < 0.01).
