# Supplementary material for: Bradykinin Preconditioning Improves Therapeutic Potential of Human Endothelial Progenitor Cells in Infarcted Myocardium
Source: PLoS One. 2013 Dec 2;8(12):e81505. doi: 10.1371/journal.pone.0081505 (PMC3846887; doi:10.1371/journal.pone.0081505)
Supplement: Results S2 — These are the results for Figure S2. (DOCX) [file pone.0081505.s004.docx]

*Efficiency of DiD labeling and correlation between cell number and fluorescence intensity*

After culturing for three passages, cells were processed for *ex vivo* optical imaging. All cells were DiD positive based on fluorescence microscopy (Figure S2A). The cell number correlated linearly with the fluorescence counts (r^2^ = 0.9977, Figures S2B and C), indicating that optical imaging is a reliable tool for assessing cell viability.
